# Pontin/Tip49 acts as a novel regulator of JNK pathway

**DOI:** 10.1038/s41419-018-0977-z

**Published:** 2018-09-24

**Authors:** Xingjun Wang, Xirui Huang, Chenxi Wu, Lei Xue

**Affiliations:** 1 Institute of Intervention Vessel, Shanghai 10th People’s Hospital, Shanghai Key Laboratory of Signaling and Diseases Research, School of Life Science and Technology, Tongji University, 1239 Siping Road, Shanghai, 200092 China; 2 Department of Neuroscience, Scripps Research Institute, Florida 130 Scripps Way Jupiter, Florida, 33458 USA; 3 College of Chinese Medicine, North China University of Science and Technology, 21 Bohai Road, Tangshan, 063210 China

Pontin/Tip49, one of the superfamily members of the AAA+ ATPases, is involved in many functions in cell contexts from invertebrates to mammals. Pontin is reported to play a role in cancer^1,2^ such as tumor invasion^3^, regulation of growth and proliferation^4^, acts as a cofactor for Oct4-dependent lincRNA expression in stem cells^5^, yet its role in cell death remains poorly understood. In this Comment, we will discuss our recently published article about Pontin as a negative regulator of Egr-induced JNK-mediated cell death, which highlights a possible relationship between ATPase and Egr/JNK^6^.

The c-jun N-terminal kinase (JNK) signaling pathway is highly conserved among species and governs diverse roles in animals, such as dorsal closure^7–9^, cell death^10^, tumor metastasis^11–13^, and progression of Alzheimer’s Disease^14^. To search additional regulators of the JNK pathway, we carried out a genetic screen in *Drosophila* for modulators of the tumor necrosis factor ortholog Eiger (Egr)-induced cell death^10^, and identified Pontin (Pont) as a negative regulator of the Egr–JNK pathway^6^. A mild expression of Egr in eye development induced weak cell death and produced a rough eye phenotype^10^, which could be enhanced to a small eye phenotype or suppressed to normal eye upon genetic modification, and thus, could serve as a powerful tool for genetic screen. We found that downregulation of *pont* by RNAi approach dramatically enhanced *GMR*>Egr-induced cell death and produced a small eye phenotype. This enhancement was confirmed in heterozygous *pont* mutants. Furthermore, depletion of *pont* induced JNK-dependent cell death and activated the expression of JNK target gene *puc* in wing development. As activation of JNK pathway in the developing thorax could induce cell death and generate a small notum phenotype, we wonder whether Pont also regulates JNK pathway in the thorax development. In line with this hypothesis, loss of *pont* in the developing thorax induced cell death and produced a small notum phenotype, which was suppressed in heterozygous mutants for *bsk* encoding the *Drosophila* JNK and *fos* encoding the AP-1 component, indicating that Pont is physiologically required to inhibit JNK-Fos-mediated cell death in thorax development. To probe how Pont regulates the JNK pathway, we checked the activity of JNK by its phosphorylation, finding that depletion of *pont* in the wing disc resulted in elevated JNK phosphorylation, which was fully suppressed by the expression of Puc, a JNK phosphatase. Thus, endogenous Pont negatively regulates JNK-mediated cell death by inhibiting the phosphorylation of JNK.

**Fig. 1:**
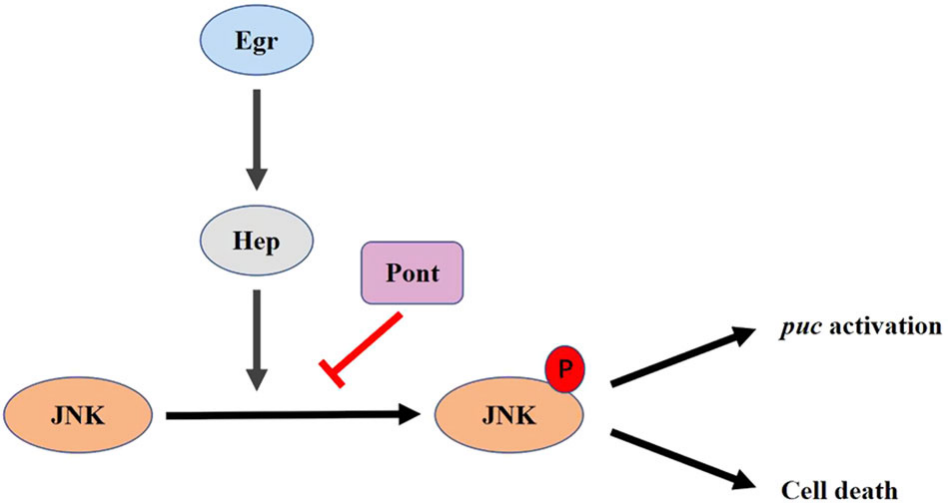
The role of Pont in the Egr–JNK pathway. An illustration of Pont in the Egr–JNK pathway is shown. Pont inhibits Hep-triggered JNK phosphorylation, which leads to cell death and target gene *puc* expression

Next, we examined if increaseed Pont is sufficient to suppress ectopic Egr-induced JNK-mediated cell death in development. We found expression of Pont suffice to block ectopic Egr-induced *puc* expression, cell death and morphological defects in the adult eye, wing and thorax, suggesting Pont inhibits Egr-triggered JNK activation and cell death in a non-tissue-specific manner. To characterize the epistasis of Pont in the Egr–JNK pathway, we checked the genetic interaction between Hep, a JNK kinase, and Pont. We found that gain of Pont compromised Hep-induced cell death phenotypes in the eye, thorax and wing, indicating Pont may act downstream of Hep. Collectively, this study highlights a novel role of Pont ATPase in the Egr–JNK pathway (Fig. 1).
